# Bullous scabies: a case report and review of the literature

**DOI:** 10.1186/s13104-015-1146-4

**Published:** 2015-06-20

**Authors:** Muhammad Arslan Arif Maan, Muhammad Soban Arif Maan, Abdul Malik Amir Humza Sohail, Muhammad Arif

**Affiliations:** Aga Khan University, Stadium Road, Karachi, Pakistan; Department of Dermatology, Punjab Medical College, Faisalabad, Pakistan

**Keywords:** Bullae, Scabies, Bullous Pemphigoid, Maculopapular rash, Pakistan

## Abstract

**Background:**

Scabies is a common parasitic infection caused by the mite *Sarcoptes Scabiei*. About 300 million cases of scabies are reported annually. Scabies usually presents clinically with an erythematous excoriated papulovesicular rash, burrows, nodules and hyperkeratotic lesions in specific body areas.

A rare presentation of scabies is the bullous pemphigoid-like bullous scabies. So far, to the best of our knowledge, only 32 cases of bullous scabies have been reported in medical literature, of which only 11 were under 60 years of age at the time of initial presentation. This is the first case of bullous scabies being reported from Pakistan.

**Case presentation:**

Herein we discuss, with reference to the existing literature, the case of a 23-year-old Punjabi male who presented with a 3 day history of a tense, non-erythematous, non-tender bulla measuring approximately 0.5 cm x 0.8 cm on the right foot near the interdigital cleft. He was diagnosed to have bullous scabies.

**Conclusion:**

The diagnosis of scabies should be considered in all patients who present with tense bullous lesions accompanied by pruritus and a maculopapular rash. This is particularly relevant if these lesions do not resolve with steroid treatment. In such patients, in order to prevent a misdiagnosis of bullous pemphigoid, scrapings for *Sarcoptes Scabiei* mites and eggs should be taken.

## Background

Scabies is a common parasitic infection caused by the mite *Sarcoptes Scabiei* [1]. It spreads through human contact; there are over 300 million cases of scabies reported annually [2].

In adults, scabies presents as a small papulovesicular rash with a clinical picture comprising of pruritus, papules and nodules associated with inflammation [3]. Subcutaneous burrows are pathognomonic of the disease and can have a widespread distribution including the flexor surfaces of the wrists, lateral aspects of fingers, elbows, anterior part of the axillary folds, the interdigital web spaces, the belt line, the scrotum in males and the areolar area of the breast in female patients [4]. The most commonly affected areas in order of incidence are the hands (86 %), wrist (82 %), genitalia of male patients (64.5 %), abdomen (56 %) and nipple area in females (28 %) [5].

Patients with scabies can develop various secondary lesions, such as folliculitis, eczema, pseudolymphoma and impetigo. Atypical presentations of scabies include urticarial rash, dermatitis herpetiformis and Darier’s disease [1].

A rare subtype of scabies is bullous scabies which occurs concurrently with, or after, the appearance of scabietic lesions. It presents as bullous pemphigoid-like bullae, and at times can even induce the formation of true bullous pemphigoid lesions [6]. The first case of bullous scabies was reported in 1974 by *Bean et al.* [7] and to the best of our knowledge only 32 cases of bullous scabies have been reported in medical literature so far [1,4,8]. Out of these, only 11 were under the age of 60 years at the time of initial presentation [1,4,8].

Herein we report the case of a 23-year-old male who presented with a bullous lesion on the right foot. He was diagnosed to have bullous scabies.

## Case presentation

A 23-year-old male belonging to the Punjabi ethnic group presented with a 3 day history of a large, tense, non-erythematous, non-tender blister on the right foot. The patient also complained of lesions on the hands for the preceding week. The lesions were pruritic (more so at night) and there was no history of fever, trauma or burns. The patient denied any history of unprotected sexual contact. His past medical and surgical history was unremarkable, as was his drug history. Family history was positive for diabetes mellitus and asthma. He had no known allergies, was a nonsmoker and did not have any addictions. Review of systems was also unremarkable.

**Fig. 1:**
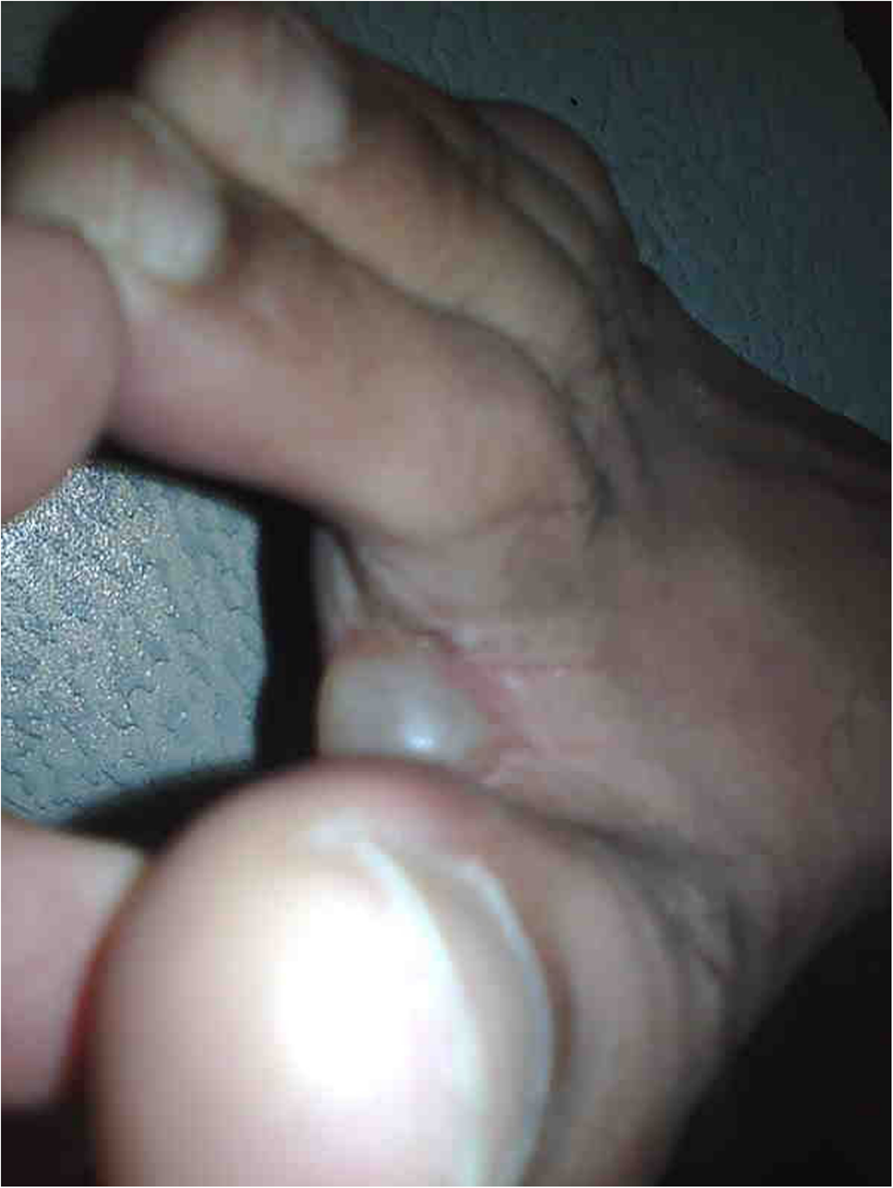
The figure shows a large, tense, non-erythematous bullous lesion, measuring approximately 0.5 cm × 0.8 cm, seen on the planter surface of the right foot near the interdigital cleft

**Fig. 2:**
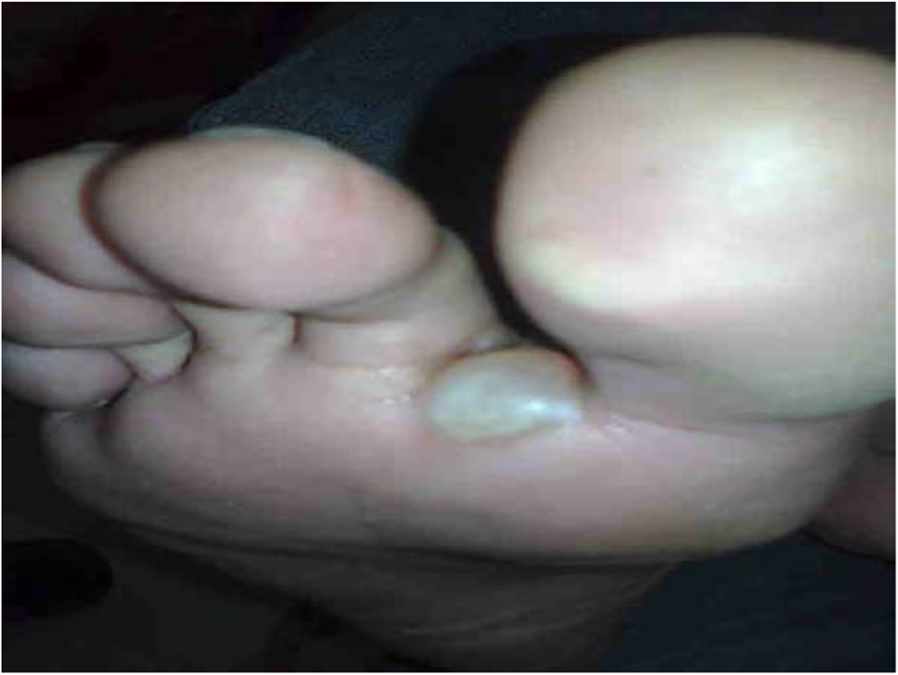
Shows another view of the same bulla

On examination, the patient was hemodynamically stable. Cutaneous examination revealed erythematous, excoriated maculopapular lesions and burrows on the wrists and the interdigital areas of both the hands. A tense bulla measuring approximately 0.5 cm x 0.8 cm was noted on the plantar surface of the right foot near the interdigital cleft (Figs. 1 and 2). It was filled with clear fluid and was negative for Nikolsky sign. There were no mucosal lesions. Systemic examination was otherwise unremarkable.

The differential diagnosis based on this initial encounter included bullous scabies, bullous impetigo, frictional bulla and bullous pemphigoid.

Scrapings were taken from the maculopapular lesions on the hands and the bulla on the foot; direct microscopy, prepared from 10 % potassium hydroxide (KOH), revealed mites and eggs of *Sarcoptes Scabiei* in scrapings from both the points. Direct swabs from the hand lesions and the bulla did not show any bacterial or fungal growth upon culture.

Based on the history, examination and investigations’ findings, the final diagnosis of bullous scabies was made.

The patient was treated with whole body applications of 5 % topical permethrin and local application of anti-histamine lotion. Complete remission of the lesions was noted after 3 weeks. The patient remained disease free for upto 18 months of follow-up.

## Discussion

Bullous scabies usually occurs in the elderly population. The cases that we came across during our review had a predominantly male predilection (21 out of 30 were males). Out of the 32 cases reviewed, most (21 out of 32) of the patients were older than 60 years. It usually presents as intensely pruritic bullae, which may be tense or flaccid and at times may have associated hemorrhage. They are usually present in scabies prone areas and may or may not have associated classic scabietic lesions [9].

The pathogenesis of bullae formation in scabies is not entirely clear and a number of theories have been proposed to explain their pathogenesis. *Staphylococcus Aureus* superinfection of mite lesions is one mechanism that can result in vesicle formation; this is similar to the bullae formation in bullous impetigo [9]. The second mechanism that has been proposed is autoantibody mediated immune damage leading to bulla formation.

Various mechanisms have been proposed for the production of these antibodies. According to one hypothesis, antibodies could be produced due to lytic destruction of the basement membrane by mites’ enzymes (leading to antigenic changes) [10]. The result of these changes is the production of BP-like antigens. These antigens will elicit BP antibodies that activate the compliment cascade, and attract eosinophils and lymphocytes; subsequent secretion of protease enzymes leads to dermoepidermal separation [10]. The presence of mites within the intraepidermal blisters further strengthens the theory that mite enzymes have a role in the pathogenesis of bullae formation [11].

Another theory suggests that the mite may elicit antibodies that cross-react with the basement membrane zone (BMZ) antigens (antigen mimicry) [10]; these antibodies (BP antibodies) act like those present in true autoimmune diseases, resulting in autoimmune damage [12]. We therefore have reason to expect antibodies to the BP antigens to be positive, as demonstrated in some cases in the literature; in one previous Western blot study, circulating antibodies against BP180 and/or BP 230 were found in the sera of two scabetic patients with bullous eruptions [13]. It also shows that at least some bullous eruptions in scabies patients are true bullous pemphigoid lesions. There are various possibilities in such cases; while the two diseases may occur simultaneously in a patient independently from each other, oftentimes the scabies is thought to trigger the characteristic bullous eruptions of bullous pemphigoid via a Koebner phenomenon. This is especially of relevance in those cases where the bullae are located exclusively on the body sites where there are scabietic lesions present as well. Moreover, in these patients where bullae appear solely on the sites affected by scabies, a possible explanation for the bullous eruptions can also be a Type 1 immune response to an antigen from the saliva of Sarcoptes Scabiei; it has been reported that the occurrence of bullous allergic hypersensitivity reactions to bug bites mediated by immunoglobulin E against compounds in the saliva of the biting insect is possible [14]. Our patient did not have any circulating antibody test, so we cannot comment regarding this aspect–pathogenesis–of bullous scabies in relation to our patient.

On direct immunofluorescence, complement component 3 (C3) and Immunoglobulin G deposition is often seen in bullous scabies. Out of the 25 cases in which direct immunoflouresence was performed, 9 had IgG deposition and C3 deposition was present in 13 cases. The pattern of IgG deposition was linear in 8 cases and granular in 3; the 2 cases reported by *Brawan, et al.* [15] showed both granular as well as linear IgG deposition in the basement membrane zone.

The various signs that have been used to confirm the diagnosis of bullous scabies include bullous eruptions over sites of the body that are usually prone to scabies, involvement of genital organs, nocturnal itch, improvement after application of antiscabietic medications and detection of the scabies mite on histopathology sections of lesions [1].

Bullous scabies is usually treated in the same way as the classical form of scabies [1]. According to The Loss Angeles County Department of Public Health Acute Communicable Disease Program, the recommended medications for scabies are 5 % permethrin cream, 10 % crotamiton lotion/cream and ivermectin [16]. Other antiscabietic agents that are employed are benzyl benzoate, malathion, and 6 % sulfur ointment [4].

In some cases of bullous scabies, a short course of oral steroids may also be required [17]. In more serious cases or in cases of steroid intolerance, other immunosuppressive agents, such as methotrexate, azathioprine, mycophenolate mofetil and cyclosporine, can be employed [4]. These may also be used as adjunct therapy. Although the mechanism of formation of bullae in bullous scabies is immune mediated, it is hard to explain why cases have been reported in which bullous scabies failed to respond to glucocorticoid, immunoglobulin and cyclophosphamide therapy [8].

Out of the 32 cases reported so far, 8 patients were treated with gamma benzene hexachloride, 7 with benzyl benzoate, 5 with permethrin, and 3 with oral ivermectin; combinations of permethrin with oral ivermectin, oral and topical steroids, gamma benzene hexachloride or sulfur were also used in some cases [1,4,8].

## Conclusion

It is suggested that the diagnosis of scabies be considered in all patients who present with tense bullous lesions accompanied by pruritis and a papular rash. This is particularly relevant if these lesions do not resolve with steroid treatment. Suspecting scabies in such patients is therefore of paramount importance, as it may not only co-exist with bullous pemphigoid, but is also an important possible precipitant for this condition. In such patients, consequently, it is important to test the lesions for *Sarcoptes Scabiei* mites and eggs.

## Consent

Written informed consent was obtained from the patient for publication of this case report and accompanying images. A copy of the written consent is available for review by the Editor-in-Chief of this journal.

## Abbreviations

BMZ: Basement membrane zone
IgG: Immunoglobulin G
C3: (Complement) Component 3
BP: Bullous pemphigoid (antigen)
KOH: Potassium hydroxide
